# Prevalence of responsible research practices among academics in The Netherlands

**DOI:** 10.12688/f1000research.110664.2

**Published:** 2022-08-08

**Authors:** Gowri Gopalakrishna, Jelte M. Wicherts, Gerko Vink, Ineke Stoop, Olmo R. van den Akker, Gerben ter Riet, Lex M. Bouter

**Affiliations:** 1 Department of Epidemiology and Data Science, Amsterdam University Medical Centers, Amsterdam, The Netherlands; 2 Department of Methodology and Statistics, Tilburg University, Tilburg, The Netherlands; 3 Department of Methodology and Statistics, Utrecht University, Utrecht, The Netherlands; 4 The Netherlands Institute for Social Research, Den Haag, The Netherlands; 5 Center of Expertise Urban Vitality, Faculty of Health, Amsterdam University of Applied Science, Amsterdam, The Netherlands; 6 Department of Philosophy, Faculty of Humanities, Vrije Universiteit Amsterdam, Amsterdam, The Netherlands

**Keywords:** Responsible conduct of research, Responsible research practices, Research integrity, Open science

## Abstract

**Background: **Traditionally, research integrity studies have focused on research misbehaviors and their explanations. Over time, attention has shifted towards preventing questionable research practices and promoting responsible ones. However, data on the prevalence of responsible research practices, especially open methods, open codes and open data and their underlying associative factors, remains scarce.

**Methods:** We conducted a web-based anonymized questionnaire, targeting all academic researchers working at or affiliated to a university or university medical center in The Netherlands, to investigate the prevalence and potential explanatory factors of 11 responsible research practices.

**Results: **A total of 6,813 academics completed the survey, the results of which show that prevalence of responsible practices differs substantially across disciplines and ranks, with 99 percent avoiding plagiarism in their work but less than 50 percent pre-registering a research protocol. Arts and humanities scholars as well as PhD candidates and junior researchers engaged less often in responsible research practices. Publication pressure negatively affected responsible practices, while mentoring, scientific norms subscription and funding pressure stimulated them.

**Conclusions: **Understanding the prevalence of responsible research practices across disciplines and ranks, as well as their associated explanatory factors, can help to systematically address disciplinary- and academic rank-specific obstacles, and thereby facilitate responsible conduct of research.

## Introduction

There has been a clear rise in publications and efforts aimed at promoting research integrity in recent years, ^1^^–^^8^ including pleas for the adoption and promotion of open science and other RRPs aimed at increasing the trustworthiness of research through increased transparency. In particular, open methods ( *e.g.* preregistration of study protocols), open codes (for data analysis), open data (following the FAIR principles ^9^) and open access (rendering publications available at no cost for users) play an important role. ^4^

A number of explanatory factors such as scientific norms subscription, fair distribution of resources, rewards and recognitions ( *i.e.* organizational justice), perceived pressures researchers face ( *e.g.* competition, work, publication and funding pressures), and support by mentors have been suggested to be important in fostering high-quality research. ^10^^–^^12^ So far however, the body of research on research integrity has focused largely on how to minimize QRPs but not so much on the empirical evidence on the adoption of foster RRPs and the associative underlying factors that may be important in encouraging the uptake of these practices. These studies typically have a narrow disciplinary scope covering few possible explanatory factors. ^10^^–^^17^

The National Survey on Research Integrity (NSRI) ^18^ was designed to take a balanced, research-wide approach to report on the prevalence of RRPs, QRPs and research misconduct, in addition to exploring the potential explanatory factors associated with these behaviors in a single survey. The NSRI targeted the entire population of academic researchers in The Netherlands, across all disciplinary fields and academic ranks.

The objectives of the NSRI were:

1) to estimate prevalence of RRPs, QRPs and research misconduct, and
2) to study the association between possible explanatory factors and RRPs, QRPs and research misconduct.

In this paper we focus on the prevalence of RRPs and the explanatory factors that may help or hinder responsible conduct of research. Elsewhere we report on QRPs, research misconduct and their associated explanatory factors. ^19^

## Methods

### Ethics approval

This study was performed in accordance with guidelines and regulations from Amsterdam University Medical Centers and the Declaration of Helsinki. In addition, the Ethics Review Board of the School of Social and Behavioral Sciences of Tilburg University approved this study (Approval Number: RP274). The Dutch Medical Research Involving Human Subjects Act (WMO) was deemed not applicable to this study by the Institutional Review Board of the Amsterdam University Medical Centers (Reference Number: 2020.286).

The full NSRI study protocol, ethics approvals, complete data analysis plan and final dataset can be found on Open Science Framework. ^20^ Below we summarize the salient study features.

### Study design

The NSRI was a cross-sectional study using a web-based anonymized questionnaire. All academic researchers working at or affiliated to at least one of 15 universities or seven university medical centers (UMCs) in The Netherlands were invited by email to participate. To be eligible, researchers had, on average, to do at least eight hours of research-related activities weekly, belong to Life and Medical Sciences, Social and Behavioural Sciences, Natural and Engineering sciences, or the Arts and Humanities, and had to be a PhD candidate or junior researcher, postdoctoral researcher or assistant professor, or associate or full professor.

The survey was conducted by a trusted third party, Kantar Public, ^21^ which is an international market research company that adheres to the ICC/ESOMAR International Code of standards. ^22^ Kantar Public’s sole responsibility was to send the survey invitations and reminders by email to our target population and send the anonymized dataset at the end of the data collection period to the research team.

Universities and UMCs that supported NSRI supplied Kantar Public with the email addresses of their eligible researchers. Email addresses for the other institutes were obtained through publicly available sources, such as university websites and PubMed.

Researchers’ informed consent was sought through a first email invitation which contained the survey link, an explanation of NSRI’s purpose and its identity protection measures. Starting the survey after this section on informed consent implied written consent. Consenting invitees could therefore immediately participate in the survey thereafter. NSRI was open for data collection for seven weeks, during which three reminder emails were sent to non-responders, at a one- to two-week interval period. Only after the full data analysis plan had been finalized and preregistered on the Open Science Framework ^20^ did Kantar Public send us the anonymized dataset containing individual responses.

### Survey instrument

NSRI comprised four components: 11 QRPs, 11 RRPs, two research misconduct questions on falsification and fabrication (FF) and 12 explanatory factor scales (75 questions). The survey started with a number of background questions to assess eligibility of respondents. These included questions on one’s weekly average duration of research-related work, one’s dominant field of research, academic rank, gender and whether one was conducting empirical research or not. ^20^

All respondents, regardless of their disciplinary field or academic rank, were presented with the same set of RRPs, QRPs and research misconduct questions on FF. These questions referred to the last three years in order to minimize recall bias. The 11 RRPs were adapted from the Dutch Code of Conduct for Research Integrity 2018 ^11^ and a survey among participants of the World Conferences on Research Integrity. ^23^ The first author of this manuscript created the initial formulations of the RRPs which covered study design, data collection, reporting, open science practices, conflicts of interest and collaboration. These 11 RRP formulations were reviewed and agreed upon in two rounds: first within the NSRI core research team, and subsequently by an external group of multidisciplinary experts who formed the NSRI Steering Committee. ^18^ All 11 RRPs had a seven-point Likert scale ranging from 1 = never to 7 = always, in addition to a “not applicable” (NA) answer option.

The explanatory factors scales were based on psychometrically tested scales in the research integrity literature and focused on action-ability. Twelve were selected: scientific norms, peer norms, perceived work pressure, publication pressure, pressure due to dependence on funding, mentoring (responsible and survival), competitiveness of the research field, organizational justice (distributional and procedural), and likelihood of QRP detection by collaborators and reviewers. ^10^^–^^12^^,^^18^^,^^23^^–^^27^ Some of the scales were incorporated into the NSRI questionnaire *verbatim*, others were adapted for our population or newly created (see *Extended data*: Table 5). With respect to the explanatory factor scale on work pressure, this can be defined as “the degree to which an academic has to work fast and hard, has a great deal to do, but with too little time” while publication pressure can be defined as the degree to which an academic has to publish in high-prestige journals in order to have a sustainable career. ^28^ *Extended data* Table 6 ^29^ provides the full list of questions we included in the questionnaire.

Face validity of the NSRI questionnaire was tested in several ways. The QRP-related questions underwent extensive focus group testing in the instrument development stage of the project. Both the QRPs and RRPs were further refined through several rounds of discussions with the core research team, with the project’s Steering Committee and with an independent expert panel set up to review the entire questionnaire. Preliminary pilot testing was conducted for some of the explanatory factor scales, listed in Extended Data Table 5 along with the results of the factor analysis (factor loadings), whereas others were re-used from validated instruments, also detailed in Table 5 ( *Extended* data). ^29^ Explanatory factor scales that are indicated as having been piloted will be reported on in future publications. In addition, internal consistency was tested and is reported as Cronbach’s Alpha in Extended Data Table 1b. Inter-rater reliability was not applicable as the survey was self-administered; however test-retest reliability was not tested. Finally, the NSRI questionnaire’s comprehensibility was pre-tested in cognitive interviews with 18 academics from different ranks and disciplines. ^30^ In summary, the comments centered around improvement in layout, such as the removal of an instructional video on the RR technique which was said to be redundant, improvement in the clarity of the instructions, and recommendations to emphasize certain words in the questionnaire by using different fonts for improved clarity. The full report of the cognitive interview can be accessed at the Open Science Framework. ^20^

We used “missingness by design” to minimize survey completion time resulting in a total of 20 minutes on average for completion, each invitee received one of three random subsets of 50 explanatory factor items from the full set of 75 (see Table 5, *Extended data* ^29^). All explanatory factor items had seven-point Likert scales. In addition, the two perceived likelihood of QRP detection scales, the procedural organizational justice scale and the funding pressure scale had a NA answer option. There was no item non-response option as respondents had to either complete the full survey or withdraw.

### Statistical analysis

We report on RRPs both in terms of prevalence and overall RRP mean. We operationalized prevalence as the proportion of participants that scored 5, 6 or 7 among the participants that deemed the RRP at issue applicable. Mean scores of individual RRPs only consider respondents that deemed the RRP to be applicable. In the multiple linear regression analysis, overall RRP mean was computed as the average score on the 11 RRPs, with the not-applicable scores recoded to 1 ( *i.e.*, “never”). Extended data: Figures 2a to 2e show the distribution of responses, including the “not-applicable” category for the 11 RRPs. ^29^ The associations of the overall RRP mean with the five background characteristics ( *Extended data*: Table 1a ^29^) and the explanatory factor scales were investigated with multiple linear regression. ^31^

For the multivariate analyses of the explanatory factor scales, we used z-scores computed as the first principal component of the corresponding items. ^32^ Missing explanatory factor item scores due to ‘not applicable’ answers were replaced by the mean z-score of the other items of the same scale. Multiple imputation with mice in R ^32^ (version 4.0.3) was employed to deal with the missingness by design. Fifty complete data sets were generated by imputing the missing values using predictive mean matching. ^33^^,^^34^ The linear regression models were fitted to each of the 50 data sets, and the results combined into a single inference. To incorporate uncertainty due to the nonresponse, the inferences were combined according to Rubin’s Rules. ^35^ All models contained all explanatory scales and the five background characteristics. The full statistical analysis plan, and analysis codes were preregistered on the Open Science Framework ^20^ including the following pre-specified subgroup analyses: field by rank, publication pressure by rank, funding pressure by rank, competition by disciplinary field, and detection (by reviewers or by collaborators) by disciplinary field.

### Identity protection

Respondents’ identities were protected in accordance with the European General Data Protection Regulations (GDPR) and corresponding legislation in The Netherlands. In addition, we had Kantar Public conduct the survey to ensure that the email addresses of respondents were never handled by the research team. Kantar Public did not store respondents’ URLs and IP addresses. Only a fully anonymized dataset was sent to the research team upon closure of data collection and preregistration of the statistical analysis plan. Finally, we conducted analyses at aggregate levels only ( *i.e.*, across disciplinary fields, gender, academic ranks, whether respondents conducted empirical research, and whether they came from NSRI supporting institutions).

## Results

### Descriptive analyses

**Figure 1.**
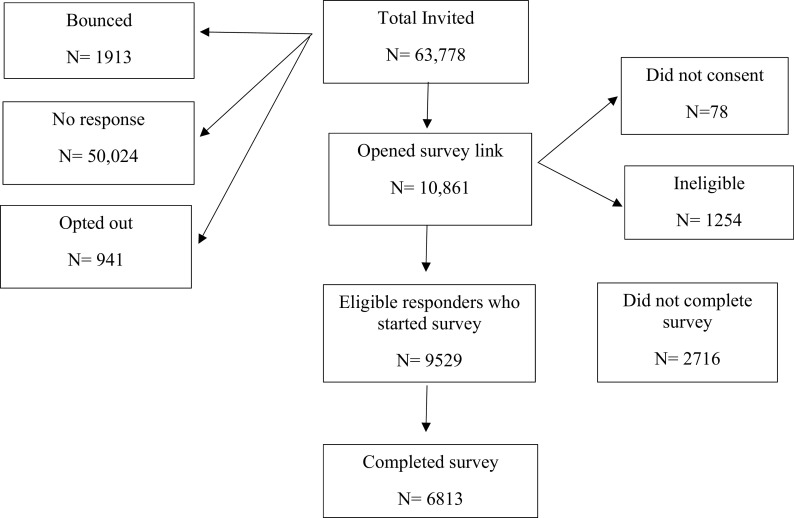
Flow chart of the survey.

A total of 63,778 emails were sent out ( Figure 1) and 9,529 eligible respondents started the survey. Of these, 2,716 stopped the survey prematurely and 6,813 completed the survey. The response could only be reliably calculated for the eight supporting institutions (Figure 1a, *Extended data* ^29^) and was 21.1%. This percentage was derived by dividing total number of eligible individuals who opened the survey invitation (4414) by the total number of individuals who were invited from the eight supporting institutions (20879). Extended Figure 1a ^29^ provides a detailed explanation of this calculation.

Extended data: Table 1a gives a breakdown of all respondents stratified by background characteristics. ^29^ Male and female respondents were fairly equally split among the respondents. For the natural and engineering sciences, women accounted for 24.9% of respondents. In the highest academic rank of associate and full professors, women made up less than 30% of respondents (Table 1a, *Extended data* ^29^). Nearly 90% of all respondents are engaged in empirical research and about half (48%) come from the eight supporting institutions. Respondents from supporting and non-supporting institutions were fairly evenly distributed across disciplinary fields and academic ranks except for the natural and engineering sciences where less than one in four (23.5%) came from supporting institutions.

Extended data: Table 1a ^29^ describes the distribution of survey respondents by gender, disciplinary field, engagement in empirical research and if they were from a supporting institution or not. *Extended data* Table 1c ^29^ describes the distribution of the explanatory factor scales by disciplinary field and academic rank. The full description of these distributions can be read at Gopalakrishna *et al.* ^19^

### Prevalence of RRPs

The five most prevalent RRPs ( *i.e.* with a Likert scale score of 5, 6 or 7) had a prevalence range of 86.4% to 99% ( Table 1; Figure 2, *Extended data* ^29^). Fair ordering of authorships (RRP 3) and preregistration of study protocols (RRP 6) showed the largest percentage differences between the Life and Medical Sciences and the Arts and Humanities (RRP 3: 75.7 vs 91.6% and RRP 6: 50.8% versus 30.2%). PhD candidates and junior researchers (74.2%) reported the lowest prevalence for RRP3 on fair allocation of authorships compared to associate and full professors (90.9%).

**Table 1.** Estimated prevalence (95% confidence intervals) of the 11 RRPs stratified by disciplinary field and academic rank.

RRP	Description (In the last three years …)	Disciplinary field				Academic rank			
		Life and medical sciences	Social and behavioral sciences	Natural and engineering sciences	Arts and humanities	PhD candidates and junior researchers	Postdocs and assistant professors	Associate and full professors	Overall
**RRP1**	I disclosed who funded my studies and all my relevant financial and non-finan1bcial interests in my publications	98.6 (98.0,99.0)	96.2 (95.1,97.0)	94.0 (92.6,95.2)	93.2 (90.3,95.3)	94.0 (92.6,95.1)	97.3 (96.6,97.9)	97.5 (96.7,98.2)	96.5 (96.0,97.0)
**RRP2**	I took steps to correct errors in my published work whenever I and/or peers provided valid reasons for such a correction	88.9 (87.1,90.5)	83.4 (80.7,85.8)	85.5 (82.9,87.8)	86.5 (82.0,90.0)	87.9 (85.5,89.9)	84.5 (82.5,86.4)	87.7 (85.6,89.6)	86.4 (85.2,87.6)
**RRP3**	The allocation and ordering of authorships in my publications, were fair and in line with the standards of my discipline	75.7 (74.0,77.3)	84.1 (82.4,85.8)	86.6 (84.7,88.3)	91.6 (88.7,93.8)	74.2 (72.1,76.3)	79.6 (78.0,81.1)	90.9 (89.5,92.1)	81.8 (80.8,82.7)
**RRP4**	I contributed, where appropriate, to making my research data findable, accessible, interoperable and reusable in accordance with the FAIR principles	74.8 (73.1,76.5)	70.7 (68.4,72.8)	77.5 (75.1,79.7)	84.6 (80.9,87.7)	75.2 (73.0,77.4)	73.6 (71.8,75.3)	76.6 (74.6,78.4)	75.0(73.9,76.1)
**RRP5**	I kept a comprehensive record of my research decisions throughout my studies.	57.2 (55.3,59.2)	56.5 (54.2,58.8)	54.0 (51.2,56.7)	57.1 (52.5,61.6)	62.2 (59.9,64.4)	56.4 (54.4,58.3)	50.4 (48.1,52.7)	56.3 (55.1,57.6)
**RRP6**	I pre-registered my study protocols in line with open science practices	50.8 (48.5,53.1)	38.9 (36.3,41.6)	31.9 (28.4,35.5)	30.2 (24.1,37.1)	44.3 (41.4,47.3)	40.0 (37.7,42.4)	45.2 (42.5,47.9)	42.8 (41.3,44.3)
**RRP7**	I managed my research data carefully by storing both the raw and processed versions for a period appropriate to my discipline and methodology used	90.9 (89.7,91.9)	88.8 (87.2,90.2)	84.5 (82.4,86.5)	82.8 (78.7,86.3)	90.8 (89.3,92)	87.9 (86.5,89.1)	86.7 (85.1,88.3)	88.4 (87.6,89.2)
**RRP8**	My research was published under open access conditions	75.1 (73.3,76.8)	72.7 (70.6,74.8)	73.7 (71.2,76.0)	59.1 (54.9,63.2)	73.8 (71.4,76.1)	72.0 (70.3,73.7)	72.6 (70.6,74.5)	72.6 (71.5,73.7)
**RRP9**	When making use of other people’s ideas, procedures, results and text in my publications, I cited the source accurately in accordance with the standards of my discipline	98.8 (98.3,99.2)	99.3 (98.8,99.6)	98.9 (98.1,99.3)	99.4 (98.2,99.8)	98.8 (98.2,99.2)	98.8 (98.3,99.1)	99.5 (99.1,99.8)	99.0 (98.7,99.2)
**RRP10**	I fully disclosed and made accessible on open science platforms my underlying data, computer codes, or syntaxes used in my research	47.4 (45.2,49.5)	41.4 (38.8,44.1)	52.7 (49.8,55.6)	53.4 (46.3,60.3)	42.4 (39.6,45.2)	47.1 (44.9,49.2)	51.0 (48.6,53.5)	47.2 (45.8,48.6)
**RRP11**	Before releasing results of my research, I meticulously checked my work to avoid errors and biases	94.3 (93.4,95.2)	94.8 (93.6,95.7)	93.6 (92.2,94.8)	94.2 (92,95.9)	94.3 (93.1,95.3)	94.4 (93.4,95.2)	94.2 (93.0,95.1)	94.3 (93.7,94.8)

Prevalence is based on the RRP at issue having a Likert score of 5, 6 or 7 among respondents that deemed the RRP at issue applicable; All figures in this table are percentages and refer to the last 3 years.

Extended data: Table 2 shows the discipline- and academic rank-specific prevalence of “not applicable” (NA) answers on the 11 RRPs. ^29^ Arts and Humanities scholars reported the highest prevalence of NA for nine out of the 11 RRPs. Similarly, across ranks, PhD candidates and junior researchers displayed the highest prevalence of NAs on nine out of the 11 RRPs.

The four open science practices had an overall prevalence ranging from 42.8% to 75%: (i) following the FAIR principles (RRP 4: 75%); (ii) Publishing open access (RRP 8: 72.6%); (iii) Providing underlying data, computer codes, or syntaxes (RRP 10: 47.2%) and (iv) Preregistration of study protocols (RRP 6: 42.8%) ( Table 1).

Surprisingly, the Arts and Humanities scholars had the highest prevalence for RRP 4 on following FAIR principles (84.6%). However, a closer look at RRP 4, reveals that this discipline also had the highest percentage of NA for RRP 4 (27.5%) ( *Extended data*: Table 2 ^29^). Life and Medical Sciences had the highest prevalence (50.8%) and the Arts and Humanities the lowest (30.2%) for preregistration of study protocols (RRP 6), where nearly 70% (67.8%) of the arts and humanities scholars rated RRP 6 as not applicable (Table 2, *Extended data* ^29^). Arts and Humanities scholars had the lowest prevalence (59.1%) and the Life and Medical Sciences the highest (75.1%) for publishing open access (RRP 8) ( Table 1).

### Regression analyses

Table 2a shows the results of the linear regression analysis for the five background characteristics while Table 2b shows the linear regression results for the explanatory factor scales.

**Table 2a.** Linear regression coefficients (95% confidence interval) of overall RRP mean score stratified by background characteristics.

		Overall RRP mean score
		Linear regression model Mean difference from reference category (95% CI)
**Disciplinary field** *Reference category: Life and medical sciences*	**Social and behavorial sciences**	**-0.15 (-0.20, -0.10)**
	Natural and engineering sciences	-0.03 (-0.09, 0.04)
	**Arts and humanities**	**-0.51 (-0.59, -0.42)**
**Academic rank** *Reference category: Postdocs and assistant professors*	**PhD candidates and junior researchers**	**-0.31 (-0.37, -0.25)**
	**Associate and full professors**	**0.08 (0.03, 0.14)**
**Gender** *Reference category: Male*	**Female**	**-0.07 (-0.12, -0.02)**
	Undisclosed	0.07 (-0.10, 0.24)
**Engaged in empirical research** *Reference category: Yes*	**No**	**-0.49 (-0.57, -0.42)**
**Institutional Support** *Reference category: No*	**Yes**	**-0.06 (-0.1, -0.01)**

Overall RRP mean score was computed as the average score on the 11 RRPs with the not applicable scores recoded to 1 (i.e. never). Model containing the five background variables and all 10 explanatory factor scales. Bold figures are statistically significant.

**Table 2b.** Linear regression coefficients (95% confidence intervals) of overall RRP mean score by explanatory factor scales.

	Overall RRP mean score
	Linear regression model Change in mean score per standard deviation increase (95 % CI)
**Work pressure**	**0.03 (0.01, 0.06)**
**Publication pressure**	**-0.05 (-0.08, -0.02)**
**Funding pressure**	**0.14 (0.11, 0.17)**
Mentoring (survival)	0.02 (-0.01,0.05)
**Mentoring (responsible)**	**0.15 (0.11, 0.18)**
Competitiveness	0.02 (-0.01, 0.05)
**Scientific norms**	**0.13 (0.10, 0.15)**
Peer norms	0.00 (-0.03, 0.03)
Organizational justice *	0.03 (0.00, 0.06)
**Likelihood of detection (collaborators)**	**0.05 (0.02, 0.08)**
Likelihood of detection (reviewers)	0.00 (-0.03, 0.03)

Overall RRP mean score was computed as the average score on the 11 RRPs with the not applicable scores recoded to 1 (i.e. never). Model containing the five background variables (see Table 2a) and all 10 explanatory factor scales.

* Two subscales (Distributional and Procedural Organizational Justice) were merged due to high correlation. Extended data: Table 4 shows the correlation of all the explanatory factor scales. Bold figures are statistically significant.

Table 2a shows that the Arts and Humanities scholars had a significantly lower overall RRP mean score (-0.51; 95% CI -0.59, -0.42). Similarly, doing non-empirical research was associated with a significantly lower overall RRP mean score (-0.49; 95% CI -0.57, -0.42). Interestingly, females had a significantly lower RRP mean score than males (-0.07; 95% CI -0.12, -0.02). Being a PhD candidate or junior researcher was associated with a significantly lower overall RRP mean (-0.31; 95% CI -0.37, -0.25).

One standard deviation increase on the publication pressure scale was associated with a significant decrease in overall RRP mean score (-0.05; 95% CI -0.08, -0.02) ( Table 2b). An increase of one standard deviation in the following five explanatory factor scales was associated with higher overall RRP mean, namely: (i) responsible mentoring (0.15; 95% CI 0.11, 0.18); (ii) funding pressure (0.14; 95% CI 0.11, 0.17); (iii) scientific norms subscription (0.13; 95% CI 0.10, 0.15); (iv) likelihood of QRP detection by collaborators (0.05; 95% CI 0.02, 0.08); and (v) work pressure (0.03; 95% CI 0.01, 0.06).

## Discussion

We found that overall RRP prevalence ranged from 42.8% to 99% with open science practices at the lower end (42.8% to 75.0%). The Arts and Humanities scholars had the lowest prevalence of preregistration of study protocols and open access publication. This disciplinary field also had the highest prevalence of NAs (nine out of the 11 RRPs), as did the PhD candidates and junior researchers. Arts and Humanities scholars, as well as PhD candidates and junior researchers, were associated with a significantly lower overall RRP mean score, as was doing non-empirical research and being female in gender.

Publication pressure was associated with lower overall RRP mean score while responsible mentoring, funding pressure, scientific norms subscription, likelihood of QRP detection by collaborators and work pressure were associated with higher RRP mean scores.

The results of our regression analysis suggest that publication pressure might lower RRPs, although the effect was modest. This finding complements what we found for QRPs, where publication pressure was associated with a higher odds of engaging frequently in at least one QRP. ^19^ These results suggest that lowering publication pressure may be important for fostering research integrity.

Our findings regarding scientific norms and peer norms subscription are noteworthy. ^10^^,^^12^ These scales have previously been validated and used in a study among 3,600 researchers of different disciplines in the United States of America. ^12^^,^^24^ In that study, respondents reported higher scientific norms subscription when asked about the norms a researcher should embrace, but they perceived the actual adherence to these norms by their peers to be lower. Our results corroborate these findings. ^12^

Previous authors have made calls to institutional leaders and department heads to pay increased attention to scientific norms subscription within their research cultures. ^12^^,^^25^ Our regression analysis findings reinforce these calls to revive subscription to the Mertonian scientific norms. ^24^

Mentoring was associated with a higher overall RRP mean score and was aligned with a similar study by Anderson *et al.* ^17^ Interestingly, a lack of proper supervision and mentoring of junior co-workers was the third most prevalent QRP respondents reported in our survey. ^19^ This finding was also reported in another recent survey among researchers in Amsterdam ^36^ which suggests that increased efforts to improve mentoring and supervision may be warranted within research institutions.

In our QRP analysis of the NSRI survey results, likelihood of detection by reviewers was significantly associated with less misconduct, suggesting that reviewers, more than collaborators, are important in QRP detection. ^37^ However, for RRPs, the reverse seems to be true: collaborators may be more important for fostering RRPs than reviewers.

To our surprise, we found that work pressure and funding pressure both had a small but significant association with higher RRP mean scores. One plausible explanation may be that adhering to RRPs requires a slower, more meticulous approach to performing research. However, given the cross sectional nature of our study, these findings do not indicate causality and must be interpreted with caution.

We found that scholars from the Arts and Humanities, as well as PhD candidates and junior researchers, reported RRPs more often as “not applicable”. We were unable to differentiate whether this is because these open science RRPs are truly not applicable or if these practices are simply not yet recognized as standard responsible practices in this discipline and rank. While it can be argued that not all open science practices, particularly those relating to the sharing of data and codes, are relevant for the non-empirical disciplines such as the Arts and Humanities, ^38^^,^^39^ practices like preregistration of study protocols, publishing open access and making sources, theories and hypotheses explicit and accessible, seem relevant for most types of research, empirical or not.

Arts and Humanities scholars reported the highest work pressure and competitiveness, and the lowest organizational justice and mentoring support. While our sample size for this disciplinary field was relatively small (n = 636), the finding of lower organizational justice in this discipline is consistent with a recent study. ^37^ Our regression analysis shows that Arts and Humanities scholars had significantly lower overall RRP mean scores as well as the highest prevalence of “not applicables” for nine out of the 11 RRPs. Research integrity efforts have largely focused on the biomedical, and social and behavioural sciences. ^40^ However, these results point to a need to better understand responsible research practices that may be disciplinary field-specific, namely to the Arts and Humanities discipline.

We found that PhD candidates and junior researchers had the lowest prevalence across all RRPs and were associated with the lowest overall RRP mean score. A recent Dutch survey of academics, as well as our own survey, point to inadequate mentoring and supervision of junior co-workers as a prevalent QRP. ^19^^,^^41^ This seems to underline a clear message: adequate mentoring and supervision of PhD candidates and junior researchers appears to be consistently lacking and may be contributing to lower prevalence of RRPs in this rank.

Women had a slightly lower, yet statistically significant, overall RRP mean score. While it has been previously reported that men engage in research misbehavior more than women, ^19^^,^^36^^,^^42^ our finding of lower RRP engagement for women has not been reported earlier and is a finding we hope to explore in the qualitative discussions planned in the next phase of our project.

The response proportion to this survey could only be reliably calculated for the eight supporting institutions and was 21.1% ( *Extended data* Figure 1a). This is within the range of other research integrity surveys. ^41^^,^^42^ Since there were no reliable numbers at the national level that match our study’s eligibility criteria. we were unable to assess our sample’s representativeness including for the five background characteristics. Despite this limitation, we believe our results to be valid as our main findings corroborate the findings of other national and international research integrity surveys. ^13^^,^^17^^,^^37^^,^^43^ Nonetheless, we wish to make clear that having solid data on the representativeness of our survey respondents in terms of our overall target population is vital. While this was unavailable at both the national level and within the supporting institutions, it is imperative that future surveys collect such data prior to the survey start.

A limitation in the analysis concerns the recoding NA answers into “never” for the multiple linear regressions. We expect our analyses reported in this manuscript to be an underestimation of the occurrence of true intentional RRPs as a result of this re-coding because our recoding of NA into “never” cannot distinguish between not committing a behavior because it is truly not applicable versus intentionally refraining from doing so. Our analyses may therefore underestimate the occurrence of true, intentional RRPs. We have studied other recodes of the NA answers and remain confident that our preregistered choice yields inferences that do not ignore the non-random distributions of the NA answers and do not violate theoretical and practical expectations about the relation between RRP and other studied practices.

The NSRI is one of the largest and most comprehensive research integrity surveys in academia to-date which looks at prevalence of RRPs and the potential associated explanatory factors in a single study across disciplinary fields and academic ranks.

## Data availability

### Underlying data and extended data

Repository: National Survey on Research Integrity Open Science Framework: https://doi.org/10.17605/OSF.IO/2K549. ^29^

This project contains the following underlying data:

• Data file 1. (NSRI Data Analysis/ GitHub:NSRI2020/NSRI (main)
• Data file 2. (ERB Ethics Approvals)
• Data file 3. (NSRI Cognitive Interview Report)
• Data file 4. (NSRI Questionnaire, Raw Dataset & Data Analysis Plan)

Data are available under the terms of the Creative Commons Attribution 4.0 International license (CC-BY 4.0).

## Authors' contributions

Conceptualization: GtR, JMW, LMB

Methodology: GG, GtR, GV, IS, JMW, LMB

Investigation: GG, JMW, OvdA

Visualization: GG, GtR, GV, IS, JMW, LMB, OvdA

Funding acquisition: GtR, LMB

Project administration: GG, LMB

Supervision: GG, GtR, LMB

Writing – original draft: GG

All authors reviewed and edited the manuscript.
