# Supplementary material for: Prevalence of responsible research practices among academics in The Netherlands
Source: F1000Res. 2022 Aug 8;11:471. Originally published 2022 Apr 28. [Version 2] doi: 10.12688/f1000research.110664.2 (PMC9478502; doi:10.12688/f1000research.110664.2)
Supplement: Peer review history

# Peer review: Reviewer response for version 2

**DOI:** 10.5256/f1000research.135530.r146812

**Published:** 2022-09-15

**Authors:** Elisabeth M. Bik

**Affiliations:** 1 Harbers Bik LLC, CA, USA

I thank the authors for addressing all comments on Version 1, and for editing the manuscript. I have no further comments and congratulate the authors with their paper.

Is the work clearly and accurately presented and does it cite the current literature?

Yes

If applicable, is the statistical analysis and its interpretation appropriate?

I cannot comment. A qualified statistician is required.

Are all the source data underlying the results available to ensure full reproducibility?

Yes

Is the study design appropriate and is the work technically sound?

Yes

Are the conclusions drawn adequately supported by the results?

Yes

Are sufficient details of methods and analysis provided to allow replication by others?

Yes

Reviewer Expertise:

Scientific integrity

I confirm that I have read this submission and believe that I have an appropriate level of expertise to confirm that it is of an acceptable scientific standard.

# Peer review: Reviewer response for version 2

**DOI:** 10.5256/f1000research.135530.r146813

**Published:** 2022-08-22

**Authors:** Dorothy Vera Margaret Bishop

**Affiliations:** 1 Department of Experimental Psychology, University of Oxford, Oxford, UK

I thank the authors for their detailed response to my initial report. 

 While I personally would prefer some different approaches to data analysis, I am happy that the authors have justified their choices, and because the data is available, this allows other readers to explore the data as they like. 

 Congratulations on an important paper in this field.

Is the work clearly and accurately presented and does it cite the current literature?

Yes

If applicable, is the statistical analysis and its interpretation appropriate?

Partly

Are all the source data underlying the results available to ensure full reproducibility?

Yes

Is the study design appropriate and is the work technically sound?

Partly

Are the conclusions drawn adequately supported by the results?

Partly

Are sufficient details of methods and analysis provided to allow replication by others?

Yes

Reviewer Expertise:

Psychology, Neuropsychology, Language, Reproducibility

I confirm that I have read this submission and believe that I have an appropriate level of expertise to confirm that it is of an acceptable scientific standard.

# Peer review: Reviewer response for version 1

**DOI:** 10.5256/f1000research.122291.r137500

**Published:** 2022-05-27

**Authors:** Dorothy Vera Margaret Bishop

**Affiliations:** 1 Department of Experimental Psychology, University of Oxford, Oxford, UK

**Summary**

 This is the 2 ^nd^ paper reporting results from the National Survey on Research Integrity in the Netherlands (NSRI), this time with a focus on frequency of, and drivers of responsible research practices (RRPs). The previous study reported on questionable research practices and a future report will consider the associations between the two. This seems a reasonable way to divide up the report of findings. The authors report that frequency of RRPs varies with discipline and they conduct a pre-registered regression analysis to look at predictors of RRPs. I evaluated this work without looking at the existing reviewer report, in order to give an independent opinion.

**Evaluation**

 This is a substantial piece of work; the survey materials that the authors have developed are impressive and they home in on key constructs for tackling issues around responsible research.  It was particularly impressive that the researchers achieved the support of many research institutions in the Netherlands. This is a rich dataset, which is openly available to other researchers, greatly enhancing its value.

 I have many suggestions for improvement, however, as I think the value and comprehensibility of the work could be enhanced. Since the F1000 model requires reviewers to give approval in order for the work to be indexed, I will note which points I see as most important to achieve that. 

**1. Response rate.**

 A major limitation of this study is the low response rate (20%). The authors mention this in the Discussion and note that their response rate is comparable to other studies of this kind. That is certainly true (in fact, it is a relatively high rate!), and I sympathise, having had similar experiences in studies of this kind I've been involved in. Nevertheless, it really limits the conclusions one can draw, especially if there is no information about how this self-selection bias affected who responded. The authors note that they cannot assess the sample's representativeness even for the five background characteristics, but "Nevertheless, we believe our results to be valid as our main finding align well with the findings of other national and international research integrity surveys". But those other surveys suffer from the same problem: self-selection bias.  Given that one of the goals of the study is to assess the prevalence, there is serious potential for biased estimates. If we have a lot of studies all with the same bias, we are in serious danger of creating illusory validity. I have two suggestions for starting to address this:

- At least for the institutions who supported the survey, gather information on the numbers of academics at the institution who fall into each discipline, and the number who fall into each academic rank. Even if these numbers are approximate, and do not describe the specific sample targeted here, they would be helpful for giving some idea about response rates in each cell of a discipline x academic rank table. Supplementary Table 1a gives some information on those completing the survey but does not actually report discipline x academic rank, which I think is an important feature (as indicated by Table 2a).  I do not see it as essential to do this, as I appreciate it may be difficult to gather this information,  but it would be very useful. If it is not possible to do it, maybe flag up the importance to gather this information upfront in future surveys.
- Discuss possible strategies for achieving better response rates in future surveys. An obvious one is incentives. I could not find any information about this in the paper, but in supplementary materials, I note the survey takes 20-25 minutes to complete. A survey that does not incentivise people to respond is going to be problematic because anyone who is busy and/or regards the subject of the survey uninteresting or irrelevant won't reply, and these people may have given different responses to those who do respond. Probably the most cost-effective way of incentivising people is to offer a lottery with one or more high-stakes prizes - e.g. enter people into a prize draw with the chance to win one of five prizes of €1000.  It's possible ethics committees would object, but I think the case for doing this is very strong - and it could be argued it is unethical to do a study that is likely to give biased findings. The most ethical solution would be to offer each respondent an adequately motivating reward (comparable to minimum wage rate) for the time spent completing the survey. With a potential pool of 60K respondents, this would get very expensive, but the research would be more valid with a smaller pool of representative academics, than with a large pool of unrepresentative people. I think some discussion of this issue, perhaps combined with some discussion of point (2) below, would be easy to incorporate in a revision and worth doing.

**2. Arts and humanities**

 On the one hand, it is good to include arts and humanities. But on the other hand, they frequently responded NA, and one can see why. Around 1/3 of respondents were not doing empirical research. The wording of questions to refer to 'open science', 'scripts', and 'data' is not ideal for this field. In my experience, academics in this area can get pretty irritated and feel they are having scientific practices imposed on them. The survey also has questions on adherence to 'scientific norms' - again, that wording is really not appropriate language for people in arts and humanities. 'Scholarly norms' would be better. Open access publishing has been a thorny issue in the humanities, especially in areas where the main output is a monograph, and there may be no funds to pay for open access. (Indeed, lack of funds for open access may be a limiting factor in other disciplines and failure to ask about that is one limitation of an otherwise very well-motivated and comprehensive survey).  My inclination would be to remove the Arts and Humanities subgroup from the analysis, as they are so very different in many respects, and I suspect the survey lacks face validity for many in those disciplines. (Of course, given that the authors have provided their data and scripts, it would be straightforward for other interested scholars to do this, so I don't insist on this as a condition for giving peer reviewer approval).

**3. Pre-registration**

 I found the pre-registration status of the paper confusing. A link, https://osf.io/2k549, is provided under Data Availability, but that refers to a Belgian pilot study. I think that is probably just an error, but it was extremely confusing and I wasted time wading through that material looking for details of the current questionnaire. Then under 'statistical analysis' I found 'The full statistical analysis plan, and analysis codes were preregistered on the Open Science Framework', and a link to an OSF page that contains data, materials, and analysis scripts, https://osf.io/ehx7q/. This material is well-organised and reasonably easy to navigate, but it does not appear to have been formally pre-registered, in the sense of having a fixed date-stamped version and I could not find a document with the data analysis plan.

 I did find data-analysis.rmd, which says "This document contains the analyses as described in NSRI data analysis plan - VERSION 7 - 20120126.docx", but I could not find that document on the OSF. Apologies if I missed it: hopefully this can be made more prominent, as this is a key aspect for evaluating the analysis. 

 This is an essential point.

**4. Skewed data.**

 For many of the items, data are skewed - in effect, these are items which amount to asking whether the respondent approves of motherhood and apple pie - everyone strongly agrees. I noticed at least one item (I did not check all), in the opposite direction, and this was one which perhaps should have been reverse scored - item F27 - most people responded 1. Some brief discussion of how this might affect results would be warranted - e.g. how does the restriction of range on some scales affect the regression coefficients?

**5. Treatment of NA responses.**

 We are told that for the regression analysis NA was coded as 1. The justification for this is questionable. My general sense is that it would be preferable to have a smaller sample for whom the survey items were valid (i.e. where NA was not used), rather than to shoehorn all respondents into an analysis which might give a misleading picture.

 It would be reassuring to readers if the analysis could be repeated by excluding all participants who responded NA, to check how this influenced findings.

 I see this as essential for clarifying the results.

**6. Difficulty in getting the sense of the main findings.**

 The underlying motivation for this work includes identification of potential explanatory factors for RRPs, presumably so interventions can be designed to modify these. Yet I could get no sense of how useful various explanatory factors would be, because data are reported largely as regression coefficients and confidence intervals, with the predictors shown on a z-score scale, which I think is derived from a principal component analysis. This would make it difficult for anyone else to use the same survey and try to replicate the results in a new sample - that would be easier if an average score from sets of items were used as an independent variable. 

 Minimally, a measure of effect size, such as percentage of variance explained, would be useful. I had to go to Supplementary Materials to find more detail of basic results of interest, and when I did that there were some anomalies - see point j below. It is a very large and complex dataset and I appreciate that the authors did not want to overwhelm readers with information. Nevertheless, I feel they have gone too far in the direction of economical presentation so that the reader has less of an immediate sense of what the results mean. This is not helped by having Methods placed after Results (see point a below).

 I was interested, for instance, in understanding more about the unexpected association between work pressure/funding pressure and RRPs. I didn't really understand the authors' explanation 'adherence to RRPs requires a slower, more meticulous approach' - I can see that might increase work pressure because there is more to do, but it wasn't so clear for funding pressure.  Why would increased funding pressure increase RRPs? Perhaps funders are these days demanding that evidence of RRPs is shown in proposals? What's interesting though is that some might leap on this finding to justify putting more pressure on researchers, with some kind of 'more pain, more gain' argument. Of course, they could be right! This gets right to the heart of research culture: in the past, many disciplines had a 'survival of the fittest' approach with ECRs - there was an implicit ethos that research was tough, that putting pressure on ECRs would select for the best researchers, with less committed researchers dropping out. If the most committed are also those who adopt new, open practices, then you might get this kind of association. I'm not advocating this as an explanation, which is completely against current ideas of nurturing ECRs to get the best from them! But it is important to get a more detailed picture of what is going on here.

 Accordingly, I wrote a little script to explore this finding and this suggests that it's a complex picture with the effect influenced by the combination of Field and Rank, as well as Empirical Research. It also looked as if the association was at least in part driven by the NA responders doing non-empirical research.  If I am able to attach figures I'll do so, but otherwise, here is the code, where the data is just the first imputed dataset, d50[[1]].

 myplot <- ggplot(mydat, aes(x=WorkPress, y=RRP_ave, color=Research)) +

   geom_point(shape=1, size=.5)+

   geom_smooth(method=lm)

 myplot<- myplot + facet_grid(Field ~ Rank)

 The plots can be viewed on Github:

https://github.com/oscci/miscellaneous/blob/master/FundPress.pdf

https://github.com/oscci/miscellaneous/blob/master/WorkPress.pdf

 Assuming my plots are accurate, I'd be very cautious about making any general claims about the impact of either Work Pressure or Publication Pressure on the adoption of RRPs.

 I don't regard it as essential for the authors to add such plots, but I would like to see some discussion of the possible variation across disciplines/ranks, and the substantive importance of the effect sizes in real life.

**More minor points**

**a. **I dislike the practice of putting Methods at the end of the paper. I see it as symptomatic of a tendency that the authors want to avoid - treating methods as less important than results. I can't make sense of the results until I have seen the methods. In fact, numerous questions occurred as I read the Results, which then were answered at the end of the paper. So please put this important material in its rightful place, after the introduction.

**b.** The file codebook.rmd does not run. The variable v is length 143, whereas descriptions and values are length 139, so they won't combine in a data frame. Ideally, we just need a document file with this information (I tried the html file, but it did not give sensibly formatted output; a simple .csv would be best).  (I'm sure this is easily fixed)

**c.** It would be good to have a table showing the breakdown of numbers in each cell formed by cross-classifying by discipline and academic rank. Some information is available on 2-way cross-classification in the supplementary tables, but it is not enough.

**d.** p 3, end of Introduction; 'associative' change to 'associated'

**e. **Mention how long the survey took to complete: I eventually found this in the online material, but it would be better in text.

**f.** p 3. It seems odd to say that there were 6813 completed surveys out of 63,778 emails and then describe a 21% response rate. I assume this reflects the fact that for nonsupporting institutions, you do not know if emails were correct?  Just a word of explanation is needed here.

**g.** Is Extended Data the same as Supplementary Materials? I assume it is and refers to the material I found at this link: https://osf.io/w9vhc/.

**h.** Why is Supplementary Table 1b not in the main text? This seems like key information from the survey, which readers will want to have readily available without needing to open supplementary documents from a link.

**i. **Similarly, I felt that to get a sense of the data, I needed to see the items corresponding to the scales shown in Supplementary Table 1b. These should be included in the main text.

**j.** Supplementary Figure 2 would also be worth including in the main text, but it needs a key indicating what each of the RRP codes is. The order of the codes seems different from the order in which each RRP is mentioned in the Tables. This figure illustrates the skew that I mentioned that affects especially Scientific Norms (I assume RRP1?) and RRP9 and RRP11. I tried to work out which scales were RRP9 and RRP11 by looking for scales with means above 5 (since most responses in Fig 2 are 6 and 7 for these scales), but there weren't any others than Scientific Norms, so this again is confusing and needs clarifying. I eventually worked it out by comparing the main paper Table 1 and the Supplementary material, but I am still confused as to why the mean scores for F9 and F11 are not higher in Supplementary Table 1b.

**k.** I recommend being more cautious in the use of causal language, e.g. talking of 'explanatory factors'. This is observational rather than experimental data, based on self-report, and it is possible that there are subject-specific factors that lead to specific kinds of responses on the 'dependent' variables and also affect reporting of 'independent' predictors.  In effect, any reporting biases by participants are confounded with both independent and dependent measures. The difficulty in assigning causality is apparent in the authors' own explanation of why work pressure predicts RRPs - this could actually be because adoption of RRPs makes more work.

 The results are still of interest but need to be reported with appropriate cautions.

Is the work clearly and accurately presented and does it cite the current literature?

Yes

If applicable, is the statistical analysis and its interpretation appropriate?

Partly

Are all the source data underlying the results available to ensure full reproducibility?

Yes

Is the study design appropriate and is the work technically sound?

Partly

Are the conclusions drawn adequately supported by the results?

Partly

Are sufficient details of methods and analysis provided to allow replication by others?

Yes

Reviewer Expertise:

Psychology, Neuropsychology, Language, Reproducibility

I confirm that I have read this submission and believe that I have an appropriate level of expertise to confirm that it is of an acceptable scientific standard, however I have significant reservations, as outlined above.

# Response

**Published:** 2022-06-24

**Authors:** Gowri Gopalakrishna

**Affiliations:** VU University Medical Center, Amsterdam, The Netherlands

*We thank the reviewer for these suggestions. We have already tried to gather data on the five background variables for the eight supporting institutions, but this proved difficult as some institutions had deleted the dataset they specially generated for our survey by the time we made this request (ie shortly after the survey collection closed in Nov 2020) or did not have this data that specifically matched the definitions we used in our survey. For example academic rank category we had a number of different ranks which were not always synonymous to how these institutions categorized their staff.*

*We have therefore chosen to address this concern of the reviewer by flagging the importance of gathering this type of information upfront for future surveys. Discussion section now includes the following sentences: “Nonetheless, we believe having solid data on the representativeness of our survey respondents in terms of our overall target population is vital. While this was unavailable at both the national level and within the supporting institutions, it is imperative that future surveys collect such data prior to the survey start.”*

**Discuss possible strategies for achieving better response rates in future surveys. An obvious one is incentives. I could not find any information about this in the paper, but in supplementary materials, I note the survey takes 20-25 minutes to complete. A survey that does not incentivise people to respond is going to be problematic because anyone who is busy and/or regards the subject of the survey uninteresting or irrelevant won't reply, and these people may have given different responses to those who do respond. Probably the most cost-effective way of incentivising people is to offer a lottery with one or more high-stakes prizes - e.g. enter people into a prize draw with the chance to win one of five prizes of €1000.  It's possible ethics committees would object, but I think the case for doing this is very strong - and it could be argued it is unethical to do a study that is likely to give biased findings. The most ethical solution would be to offer each respondent an adequately motivating reward (comparable to minimum wage rate) for the time spent completing the survey. With a potential pool of 60K respondents, this would get very expensive, but the research would be more valid with a smaller pool of representative academics, than with a large pool of unrepresentative people. I think some discussion of this issue, perhaps combined with some discussion of point (2) below, would be easy to incorporate in a revision and worth doing.**

*Author Response: Thank you for raising this relevant point. We did explore with our international advisors and steering committee of the NSRI the issue of incentives. However, we decided against this for several reasons. Firstly, the literature (a selection of which is provided below this response) and our experts we consulted were divided on the usefulness of incentives to higher the response rate. Secondly, incentivizing would likely not adequately address the issue of selection bias and may even possibly exacerbate it. There is evidence suggesting if incentives are to be used, they should be offered to all participants, unconditionally in order to be effective which in our study was not feasible due to the sample size. Finally, because of our strict privacy measures, handing out incentives to responders only was not a feasible option as we didn’t know who the responders were. We, therefore, chose to focus our efforts to increase participation by 1. drafting an attractive, yet succinct personalized invitation letter to each of the 63,778 participants in our target group 2. clearly outlining the importance of this survey, and how its outcome would help shape the research integrity climate for Netherlands and beyond, 3.ensuring strict privacy protection measures in the design of the survey (detailed in our methods section), 4. developing a reasonably short, easy to answer questionnaire suitable for a laptop and other handheld devices, 5.testing the online survey layout and usability through cognitive testing, 6.conducting a broad media campaign consisting of advertising the survey on social media and news outlets (university newsletters, national newspapers, national and international academic news magazines, personalized email invitations on a last name basis), 7.sending 3 reminders over a seven week period.*

*Literature:*

*Göritz, Anja. (2006). Incentives in Web Studies: Methodological Issues and a Review. International Journal of Internet Science. 1;*

*Göritz, A. S., & Neumann, B. P. (2016). The longitudinal effects of incentives on response quantity in online panels. Translational Issues in Psychological Science, 2(2), 163–173. https://doi.org/10.1037/tps0000071*

*Suzer-Gurtekin, Z. Tuba, Mahmoud ElKasabi, Mingnan Liu, James M. Lepkowski, Richard Curtin, and Rebecca McBee. 2016. “Effect of a Pre-Paid Incentive on Response Rates to an Address-Based Sampling (ABS) Web-Mail Survey.” Survey Practice 9 (4). https://doi.org/10.29115/SP-2016-0025.*

*Evans, J.R. and Mathur, A. (2018), "The value of online surveys: a look back and a look ahead", Internet Research, Vol. 28 No. 4, pp. 854-887. https://doi.org/10.1108/IntR-03-2018-0089*

 2. Arts and humanities

**On the one hand, it is good to include arts and humanities. But on the other hand, they frequently responded NA, and one can see why. Around 1/3 of respondents were not doing empirical research. The wording of questions to refer to 'open science', 'scripts', and 'data' is not ideal for this field. In my experience, academics in this area can get pretty irritated and feel they are having scientific practices imposed on them. The survey also has questions on adherence to 'scientific norms' - again, that wording is really not appropriate language for people in arts and humanities. 'Scholarly norms' would be better. Open access publishing has been a thorny issue in the humanities, especially in areas where the main output is a monograph, and there may be no funds to pay for open access. (Indeed, lack of funds for open access may be a limiting factor in other disciplines and failure to ask about that is one limitation of an otherwise very well-motivated and comprehensive survey).  My inclination would be to remove the Arts and Humanities subgroup from the analysis, as they are so very different in many respects, and I suspect the survey lacks face validity for many in those disciplines. (Of course, given that the authors have provided their data and scripts, it would be straightforward for other interested scholars to do this, so I don't insist on this as a condition for giving peer reviewer approval).**

*Author Response: We thank the reviewer for this valuable comment. The reviewer is right in pointing out that certain terms used in the survey may be less applicable to the Arts and Humanities including certain Open Science practices such as open access for specific fields and open sharing of data, syntaxes and codes. However, we wish to point out that many of the RRPs (about 7 of the 11) are not exclusionary to the Arts and Humanities for example RRP 2 “ I took steps to correct errors in my published work whenever I and/or peers provided valid reasons for such a correction”, RRP 5 “I kept a comprehensive record of my research decisions throughout my studies”, RRP 9 “When making use of other people’s ideas, procedures, results and text in my publications, I cited the source accurately in accordance with the standards of my discipline”. We did strive to have face validity of our survey instrument by including a sample of researchers from all four disciplinary fields including the Arts and Humanities in our focus group sessions, in the cognitive testing of the survey instrument (see Methods section, subheading survey instrument) and in our Steering Committee. Whilst we agree that some of the RRPs may truly be not applicable to this discipline, this would not be the case for the majority of the RRPs studied. Because this field is especially understudied in the field of research integrity, we feel it is all the more important to not exclude this group from our results but rather prompt a debate on why this discipline may be so different and that we need greater understanding of this discipline in the research integrity, responsible research and open science debates. Excluding this group from our analysis and study will not help the dialogue on the need for better understanding of the challenges and knowledge production methods in this discipline.*

 3. Pre-registration

**I found the pre-registration status of the paper confusing. A link, https://osf.io/2k549, is provided under Data Availability, but that refers to a Belgian pilot study. I think that is probably just an error, but it was extremely confusing and I wasted time wading through that material looking for details of the current questionnaire. Then under 'statistical analysis' I found 'The full statistical analysis plan, and analysis codes were preregistered on the Open Science Framework', and a link to an OSF page that contains data, materials, and analysis scripts, https://osf.io/ehx7q/. This material is well-organised and reasonably easy to navigate, but it does not appear to have been formally pre-registered, in the sense of having a fixed date-stamped version and I could not find a document with the data analysis plan.**

**I did find data-analysis.rmd, which says "This document contains the analyses as described in NSRI data analysis plan - VERSION 7 - 20120126.docx", but I could not find that document on the OSF. Apologies if I missed it: hopefully this can be made more prominent, as this is a key aspect for evaluating the analysis. This is an essential point.**

*Author Response: Thank you for your meticulous checking of the links provided under Data availability.  We have corrected the link under Data Availability with the correct link:* *https://osf.io/dp6zf/* *.This should have been the correct link listed which when clicked takes you to several subfolders. The data analysis plan can be found under the subfolder:  “NSRI Data Analysis > OSF Storage (United States)>NSRI Questionnaire, Raw Data & NSRI Data Analysis Plan. The NSRI Data Analysis Plan is a pdf document which is time stamped as 2021-08-03 03:11PM.*

 4. Skewed data.

**For many of the items, data are skewed - in effect, these are items which amount to asking whether the respondent approves of motherhood and apple pie - everyone strongly agrees. I noticed at least one item (I did not check all), in the opposite direction, and this was one which perhaps should have been reverse scored - item F27 - most people responded 1. Some brief discussion of how this might affect results would be warranted - e.g. how does the restriction of range on some scales affect the regression coefficients?**

*Author Response: All scales have been recoded - when applicable - such that they measure in the same direction. In the case for F27 which measures the question: “Publication pressure sometimes leads me to cut corners.” Here, most respondents did respond with a “1” indicating “never” reflecting that most respondents did not tend to favor cutting corners. On skewness and its probably effect on coefficients, this was carefully checked by our statisticians to ensure skewness did not affect our regression coefficients.*

 5. Treatment of NA responses.

**We are told that for the regression analysis NA was coded as 1. The justification for this is questionable. My general sense is that it would be preferable to have a smaller sample for whom the survey items were valid (i.e. where NA was not used), rather than to shoehorn all respondents into an analysis which might give a misleading picture.**

**It would be reassuring to readers if the analysis could be repeated by excluding all participants who responded NA, to check how this influenced findings.**

**I see this as essential for clarifying the results.**

*Author Response: Thank you for this comment. We wish to clarify that the “not applicable” values are bonafide missing values. While we understand removing them may seem semantically intuitive, there are valid statistical and procedural reasons why we chose to replace these values with the lowest observed category (1 = Never). Please allow us to explain these here:*

*First, the recoding of “not applicable” to 1 is part of our pre-registered data analysis plan. Second, we did run extensive sensitivity analyses to study the validity of our pre-registered choice. Based on these analyses we concluded that our pre-registered choice is the most valid solution to this issue in this data set i.e. we deliberately chose recoding NAs into 1 as we know the direction of any potential bias: it underestimates the true effect, thereby limiting the statistical power of our analysis. Despite this we still found some effects.These sensitivity analyses can be found in the OSF data analysis folder> subfolder entitled “Figures and Tables> Table 3 Regressions”: https://osf.io/ehx7q/.*

*Third, replacing “not applicable” with the value 0 is not a bonafide value that could have been observed. Using non-bonafide constants to fill in missing values is unreliable and statistically invalid. As such filling in zero would underestimate any parameters to a much greater extent than using a bonafide observed value would. We believe inducing such deliberate bias would therefore be undesirable. Fourth, coding “not applicables” as zero yields a positive correlation between QRP and RRP in a confirmatory factor analysis. This is counterintuitive and not in line with theoretical, nor practical expectations. Coding the “not applicables” as 1 (or any other bonafide observed value, for that matter) yields an expected negative correlation between the factors QRP and RRP.*

*Lastly, the validity of our pre-registered data analysis plan with respect to the “not applicable” has been confirmed by two independent replications on two different data structures reference: De Koning and Van der Sluis (2021). Modeling not applicable answers when data are incomplete. Master Thesis. Utrecht University.*

*To ensure this is also explained in the Limitations section of the manuscript, we have included this change in the Discussion section:*

*“We have studied other recodes of the NA answers and remain confident that our preregistered choice yields inferences that do not ignore the non-random distributions of the NA answers and do not violate theoretical and practical expectations about the relation between RRP and other studied practices.”*

 6. Difficulty in getting the sense of the main findings.

**The underlying motivation for this work includes identification of potential explanatory factors for RRPs, presumably so interventions can be designed to modify these. Yet I could get no sense of how useful various explanatory factors would be, because data are reported largely as regression coefficients and confidence intervals, with the predictors shown on a z-score scale, which I think is derived from a principal component analysis. This would make it difficult for anyone else to use the same survey and try to replicate the results in a new sample - that would be easier if an average score from sets of items were used as an independent variable.**

**Minimally, a measure of effect size, such as percentage of variance explained, would be useful. I had to go to Supplementary Materials to find more detail of basic results of interest, and when I did that there were some anomalies - see point j below. It is a very large and complex dataset and I appreciate that the authors did not want to overwhelm readers with information. Nevertheless, I feel they have gone too far in the direction of economical presentation so that the reader has less of an immediate sense of what the results mean. This is not helped by having Methods placed after Results (see point a below).**

**I was interested, for instance, in understanding more about the unexpected association between work pressure/funding pressure and RRPs. I didn't really understand the authors' explanation 'adherence to RRPs requires a slower, more meticulous approach' - I can see that might increase work pressure because there is more to do, but it wasn't so clear for funding pressure.  Why would increased funding pressure increase RRPs? Perhaps funders are these days demanding that evidence of RRPs is shown in proposals? What's interesting though is that some might leap on this finding to justify putting more pressure on researchers, with some kind of 'more pain, more gain' argument. Of course, they could be right! This gets right to the heart of research culture: in the past, many disciplines had a 'survival of the fittest' approach with ECRs - there was an implicit ethos that research was tough, that putting pressure on ECRs would select for the best researchers, with less committed researchers dropping out. If the most committed are also those who adopt new, open practices, then you might get this kind of association. I'm not advocating this as an explanation, which is completely against current ideas of nurturing ECRs to get the best from them! But it is important to get a more detailed picture of what is going on here.**

*Author Response: Thank you for raising this valid important concern. We agree that these findings must be interpreted with caution given the cross sectional nature of our study. We have emphasized this by including a sentence in the Discussion section on this topic which reads as follows “However, given the cross sectional nature of our study, these findings do not indicate causality and must be interpreted with caution”*

**Assuming my plots are accurate, I'd be very cautious about making any general claims about the impact of either Work Pressure or Publication Pressure on the adoption of RRPs.**

**I don't regard it as essential for the authors to add such plots, but I would like to see some discussion of the possible variation across disciplines/ranks, and the substantive importance of the effect sizes in real life.**

*Author Response: Thank you for this thorough analysis on this issue, However, please allow us to explain why the proposed procedure may be cause for concern. Our analysis are based on the pooled inferences obtained after multiple imputation. We use Rubin’s rules (See reference 42, pp. 76) to properly combine parameter estimates and correctly calculate the corresponding variances. Looking at a single imputed data set and interpreting the effects of that set can be dangerous and may lull the analyst into a false sense of discovery . For example, re-running the code snippet on the next imputed data set yields different plots, with different trends and interactions. This is indicative of the sampling and missing data uncertainty in the analysis problem. Rubin’s rules take the maximum likelihood estimate over the imputed sets and increase the variance about the estimates (i.e. the square of the standard error) accordingly. The procedure we followed ensures that the variability and likelihood of the obtained inference after pooling is properly incorporating the missing data and parameter uncertainty associated with the problem. Having said this, we do agree that making general claims that may imply causality is to be avoided. In that light we have included the following sentence at the end of our Discussion section as follows: “Even so, it is important to emphasize that this is a cross-sectional study and therefore does not imply causality.”*

**More minor points**

  

- **I dislike the practice of putting Methods at the end of the paper. I see it as symptomatic of a tendency that the authors want to avoid - treating methods as less important than results. I can't make sense of the results until I have seen the methods. In fact, numerous questions occurred as I read the Results, which then were answered at the end of the paper. So please put this important material in its rightful place, after the introduction.**
*Author Response: We have moved Methods such that it appears after Introduction.*
- **The file codebook.rmd does not run. The variable v is length 143, whereas descriptions and values are length 139, so they won't combine in a data frame. Ideally, we just need a document file with this information (I tried the html file, but it did not give sensibly formatted output; a simple .csv would be best).  (I'm sure this is easily fixed)**
*Author Response:* *This discrepancy in length is now fixed and can be viewed here:* *https://osf.io/2yhpq/*

- **It would be good to have a table showing the breakdown of numbers in each cell formed by cross-classifying by discipline and academic rank. Some information is available on 2-way cross-classification in the supplementary tables, but it is not enough.**
*Author Response: We have included an extra Supplementary Table 1b showing the proportion of respondents by discipline and rank in the supplementary files which can be directly viewed here https://osf.io/wju6e/*
- **p 3, end of Introduction; 'associative' change to 'associated'**
*Author Response:* *Thank you. We have made this change.*
- **Mention how long the survey took to complete: I eventually found this in the online material, but it would be better in text.**
*Author Response:* *We have included the following sentence in Methods: “We used “missingness by design” to minimize survey completion time resulting in a total of 20 minutes on average for completion”*
- **p 3. It seems odd to say that there were 6813 completed surveys out of 63,778 emails and then describe a 21% response rate. I assume this reflects the fact that for non-supporting institutions, you do not know if emails were correct?  Just a word of explanation is needed here.**
*Author Response:* *We have included a reference to Supplementary Figure 1a which provides the reader with a full and detailed explanation on how we derived the 21% response proportion. *
*The changes made now read as follows: “This percentage was derived by dividing total number of eligible individuals who opened the survey invitation (4414) by the total number of individuals who were invited from the eight supporting institutions (20879). Extended Figure 1a ^20^ provides a detailed explanation of this calculation”*
-  Is Extended Data the same as Supplementary Materials? I assume it is and refers to the material I found at this link: https://osf.io/w9vhc/.
*Author Response:* *Yes, this is correct. Extended Data is how F1000 refers to supplementary materials.*
*  *
- **Why is Supplementary Table 1b not in the main text? This seems like key information from the survey, which readers will want to have readily available without needing to open supplementary documents from a link.**
*Author Response: In our previous submission of this work to a different journal, there were restrictions on the number of tables and figures allowed in the main text as well as word limits, hence we had to make choices on the most important Tables and Figures and results to discuss. As part of this we moved Table 1b to extended data which we prefer to leave as is given F1000 whilst not having a Figures and Table limit does have a word limit which we are already at.*
- **Similarly, I felt that to get a sense of the data, I needed to see the items corresponding to the scales shown in Supplementary Table 1b. These should be included in the main text.**
*Given the length of our manuscript, including Supplementary 1b which is already rather large to also now show all items per scale we felt would make the Table even larger and cumbersome. We, therefore, included it as a supplementary table.As for the items in each scale, these are presented  Supplementary Table 5 to make it easier for the reader to digest all the information we have included in the entire manuscript.*
- **Supplementary Figure 2 would also be worth including in the main text, but it needs a key indicating what each of the RRP codes is. The order of the codes seems different from the order in which each RRP is mentioned in the Tables. This figure illustrates the skew that I mentioned that affects especially Scientific Norms (I assume RRP1?) and RRP9 and RRP11. I tried to work out which scales were RRP9 and RRP11 by looking for scales with means above 5 (since most responses in Fig 2 are 6 and 7 for these scales), but there weren't any others than Scientific Norms, so this again is confusing and needs clarifying. I eventually worked it out by comparing the main paper Table 1 and the Supplementary material, but I am still confused as to why the mean scores for F9 and F11 are not higher in Supplementary Table 1b.**
*Author Response:* *We wish to clarify that the eleven RRPs (dependent variables) shown in Supplementary Figure 2 are NOT the explanatory factor scales (independent variables). It appears that perhaps there was some confusion in the review with RRPs no. 1-11 (as shown in supplementary Figure 2) and with the 10 explanatory factors scales shown in supplementary Table 1b.Scientific Norm Subscription for example which the reviewer makes reference to is not an RRP but one of the 10 explanatory factor variables. Figure 2 show the distribution of respondent answers on the Likert answer scale and not of the explanatory factor scales.*
- I recommend being more cautious in the use of causal language, e.g. talking of 'explanatory factors'. This is observational rather than experimental data, based on self-report, and it is possible that there are subject-specific factors that lead to specific kinds of responses on the 'dependent' variables and also affect reporting of 'independent' predictors.  In effect, any reporting biases by participants are confounded with both independent and dependent measures. The difficulty in assigning causality is apparent in the authors' own explanation of why work pressure predicts RRPs - this could actually be because adoption of RRPs makes more work.
*Author Response:* *Thank you for this comment. We have included a sentence at the end of the Discussion to this effect which reads as follows: “Even so, it is important to emphasize that this is a cross-sectional study and therefore does not imply causality.”*

# Peer review: Reviewer response for version 1

**DOI:** 10.5256/f1000research.122291.r136272

**Published:** 2022-05-16

**Authors:** Elisabeth M. Bik

**Affiliations:** 1 Harbers Bik LLC, CA, USA

This manuscript is the second publication derived from a large questionnaire sent out to universities and academic medical centers in the Netherlands. While the first paper (PLOS ONE 2022, DOI: 10.1371/journal.pone.0263023; henceforth: "PONE22" ^1^) focused on questions and answers related to questionable research practices (QRP) and research misconduct, this manuscript reports on the survey part related to responsible research practices (RRP).

 This study found a negative correlation between pressure to publish and RRP, whereas its counterpart PONE22 found a positive correlation between publication pressure and QRP. Both findings are important because they strongly suggest that the increasing pressure put on scholars to publish might lead to less RRP and more QRP and science misconduct. In addition, the paper finds a correlation between the lack of adequate mentoring of junior researchers and a lower prevalence of RRPs.

 The two papers nicely complement each other, but since they both refer to the same questionnaire, there is a lot of textual and data overlap. One might argue that keeping both parts of the study together in one publication would have been a better strategy because by splitting up these papers, parts of this manuscript read as redundant and not novel. While in some cases - such as in the methods - this might be acceptable, some other parts - such as the introduction and results - should be checked for identical or very similar phrases.  Some specific examples of such similarities are noted below.

 Strengths of this paper are the scale and anonymity of the survey, the focus on both good as well as bad practices (in combination with the PONE22 paper), and the analysis of multiple science disciplines and influencing factors. This paper nicely builds upon and extends on previous surveys on research integrity and misconduct, and offers a view of the factors that drive researchers to choose between good or bad practices. This study and its PONE22 companion could and should be used for future roadmaps on how to best structure academic research.

 Specific comments

1. Introduction: the first two sentences are (nearly) identical to those of PONE22. Could they be worded a bit differently?
2. Results - "A total of 63,778 emails were sent out (Figure 1) and 9,529 eligible respondents started the survey." Figure 1 is the same as in PONE22 - the authors could just leave this sentence and add reference 19 for more details.
3. Results. "The response could only be reliably calculated for the eight supporting institutions (Figure 1a, Extended data20) and was 21.1%." - This sentence was not clear. Does this refer to the institutions (8 out of 22 invited institutions) that provided the email addresses, as mentioned in PONE22? Does the 21.1% refer to the number of invited vs completed surveys or institutions? A similar statement was found in PONE22, but there it was 21.2%.
4. The whole first part of the results "Descriptive analyses" seems nearly identical to that in PONE22. To prevent a partial duplication of already published results, it might be better to just give a short summary here, and refer to reference 19.
5. Results: Table 1: In the description of RRP1, check "finan1bcial"
6. Results. In the paragraph starting with "The four open science practices" perhaps chance 75% to 75.0% to match the number of digits to the other reported percentages.
7. Discussion. The sentence starting with "In our QRP analysis of the NSRI survey results.." refers to an older survey, reference 24. Should this not be reference 19, PONE22? Perhaps the authors could check.
8. Discussion. Could the authors perhaps define the difference between publication pressure and work pressure? I was not sure what was meant by these or how they were related to questions in the survey. This is particularly relevant since these two factors had opposite effects on RRPs. Perhaps this should be better defined in the results or here in the discussion?
9. Discussion: The last paragraphs, starting with "The email addresses of researchers..." are nearly identical to those in PONE22. Could the authors please rewrite these?
10. Methods: Again, large chunks of the text here are similar to those in PONE22. I am not sure how much textual similarity in the Methods would be acceptable for F1000, but it might still be needed to partially rewrite these and refer to reference 19.
11. Methods: Could the authors check the part starting with " The explanatory factors scales were based..."? The second sentence seems to talk specifically about QRPs, and might not be applicable for this paper.
12. This is very minor, but the text switched between "behavior" and "behaviour" and its derivatives.

Is the work clearly and accurately presented and does it cite the current literature?

Yes

If applicable, is the statistical analysis and its interpretation appropriate?

I cannot comment. A qualified statistician is required.

Are all the source data underlying the results available to ensure full reproducibility?

Yes

Is the study design appropriate and is the work technically sound?

Yes

Are the conclusions drawn adequately supported by the results?

Yes

Are sufficient details of methods and analysis provided to allow replication by others?

Yes

Reviewer Expertise:

Scientific integrity

I confirm that I have read this submission and believe that I have an appropriate level of expertise to confirm that it is of an acceptable scientific standard, however I have significant reservations, as outlined above.

## References

[1] : Prevalence of questionable research practices, research misconduct and their potential explanatory factors: A survey among academic researchers in The Netherlands. *PLoS One* .2022;17(2) : 10.1371/journal.pone.0263023 e0263023 10.1371/journal.pone.0263023 35171921PMC8849616

# Response

**Published:** 2022-06-24

**Authors:** Gowri Gopalakrishna

**Affiliations:** VU University Medical Center, Amsterdam, The Netherlands

*We thank the reviewer for these comments. With respect to the similarities between both manuscripts, we have modified parts of the Introduction, Results and Discussion sections to emphasize the differences between this manuscript and PONE22 more. The specific changes are outlined in detail below against each of the reviewer comments.*

*As the reviewer correctly points out both manuscripts, this and PONE22, are complementary to each other. Whilst the PONE22 article reports on the fabrication and falsification (FF) and questionable research practices (QRPs), this manuscript presents the responsible research practice questions (RRPs) from the National survey on Research Integrity (NSRI) and the association between each explanatory factor and the overall RRP mean score. Because both PONE22 and this manuscript report on data of the NSRI the Methods sections are indeed quite similar.*

*We did consider the option of combining both papers into one but decided against this for the following reasons:*

1. *Much of the research integrity literature has focused on negative research behaviours (i.e. QRPs and misconduct) and hardly on the empirical evidence around RRPs and the factors that may be associated with them. Combining both papers may dilute the emphasis on the RRPs which we felt warranted in line with the discussions that increasingly center around rewarding responsible research practices and open science.*
2. *Combining both papers into one would have complicated a focused discussion considerably given the large number of descriptive as well as regression-based findings. We also believed that two separate papers with clear cross-references would make the NSRI findings more assessable than everything piled in one lengthy piece.*
*For descriptive results on the explanatory factors which are similar in both papers, we have now made a reference to PONE22 in this manuscript to avoid repetition.*

**Specific comments**

**Introduction: the first two sentences are (nearly) identical to those of PONE22. Could they be worded a bit differently?**

*Author Response: Thank you for this comment. We have changed the introduction such that it now begins with the following paragraph*

*“There has been a clear rise in publications and efforts aimed at promoting research integrity in recent years, including pleas for the adoption and promotion of open science and other RRPs aimed at increasing the trustworthiness of research through increased transparency. In particular, open methods (e.g. preregistration of study protocols), open codes (for data analysis), open data (following the FAIR principles) and open access (rendering publications available at no cost for users) play an important role”*

**Results - "A total of 63,778 emails were sent out (Figure 1) and 9,529 eligible respondents started the survey." Figure 1 is the same as in PONE22 - the authors could just leave this sentence and add reference 19 for more details.**

*Author Response: Thank you for this comment. While we agree this part of the results is the same as in PONE22, we feel it is important to explicitly mention these results so a reader can immediately have the response proportion of our survey without needing to look up this important information in another publication.*

**Results. "The response could only be reliably calculated for the eight supporting institutions (Figure 1a, Extended data20) and was 21.1%." - This sentence was not clear. Does this refer to the institutions (8 out of 22 invited institutions) that provided the email addresses, as mentioned in PONE22? Does the 21.1% refer to the number of invited vs completed surveys or institutions? A similar statement was found in PONE22, but there it was 21.2%.**

*Author Response: Thank you for spotting this error in the decimal point. The figure should be 21.1% and refers to the same percentage explained in Figure 1a of the extended data reference 20. This is derived by dividing total number of eligible individuals who opened the survey invitation (4414) by the total number of individuals who were invited from the 8 supporting institutions. We have further clarified by this by changing the following sentence to read as:*

*“The response could only be reliably calculated for the eight supporting institutions (Figure 1a, Extended data20) and was 21.1%. This percentage was derived by dividing total number of eligible individuals who opened the survey invitation (4414) by the total number of individuals who were invited from the eight supporting institutions (20,879).”*

**The whole first part of the results "Descriptive analyses" seems nearly identical to that in PONE22. To prevent a partial duplication of already published results, it might be better to just give a short summary here, and refer to reference 19.**

*Author Response: We have replaced this first paragraph as suggested with the following sentence:*

*Extended Data Table 1a describes the distribution of survey respondents by gender, disciplinary field, engagement in empirical research and if they were from a supporting institution or not. Extended Data Table 1b describes the distribution of the explanatory factor scales by disciplinary field and academic rank. The full description of these distributions can be read at Gopalakrishna et al19 .*

**Results: Table 1: In the description of RRP1, check "finan1bcial"**

*Author Response: This should have been “financial” and is now corrected.*

**Results. In the paragraph starting with "The four open science practices" perhaps change 75% to 75.0% to match the number of digits to the other reported percentages.**

*Author Response:  We have changed the percentage to read as 75.0%*

**Discussion. The sentence starting with "In our QRP analysis of the NSRI survey results.." refers to an older survey, reference 24. Should this not be reference 19, PONE22? Perhaps the authors could check.**

*Author Response: Thank you for spotting this mistake. The reference should be 19 as mentioned and is now corrected.*

**Discussion. Could the authors perhaps define the difference between publication pressure and work pressure? I was not sure what was meant by these or how they were related to questions in the survey. This is particularly relevant since these two factors had opposite effects on RRPs. Perhaps this should be better defined in the results or here in the discussion?**

*Author Response: We now make this distinction more clearly in the Methods section where we have included the following sentences:*

*“With respect to the explanatory factor scale on work pressure, this can be defined as “the degree to which an academic has to work fast and hard, has a great deal to do, but with too little time” while publication pressure can be defined as the degree to which an academic feels s/he has to publish in high-prestige journals in order to have a sustainable career (Karasek & Theorell, 1990),” Extended data Table 620 provides the full list of questions we included in the questionnaire.”*

**Discussion: The last paragraphs, starting with "The email addresses of researchers..." are nearly identical to those in PONE22. Could the authors please rewrite these?**

*Author Response: We have modified these paragraphs as requested. They now read as follows:*

*“The response proportion for this survey could only reliably be calculated for the eight supporting institutions and this was 21.1%19. This is within the range of other research integrity surveys 24,30. Since there were no reliable numbers at the national level that match our study’s eligibility criteria, we were unable to assess our sample’s representativeness including the five background characteristics. Despite this limitation, we believe our results to be valid as our main findings corroborate the findings of other national and international research integrity surveys 12,17,22,24,31.*

*A limitation in our analysis concerns the recoding of the NA answers into “never” for the multiple linear regressions. We expect our analyses reported in this manuscript to be an underestimation of the occurrence of true intentional RRPs as a result of this re-coding. Because our recoding of NA into “never” cannot distinguish between not committing a behavior because it is truly not applicable versus intentionally refraining from doing so, our analyses may therefore underestimate the occurrence of true, intentional RRPs.*

*The NSRI is the one of the largest  and most comprehensive research integrity surveys in academia to-date which to study prevalence of RRPs and the potential explanatory factors that may be associated with these behaviours in a single study across disciplinary fields and academic ranks.”*

**Methods: Again, large chunks of the text here are similar to those in PONE22. I am not sure how much textual similarity in the Methods would be acceptable for F1000, but it might still be needed to partially rewrite these and refer to reference 19.**

*Author Response: Thank you for this comment. We feel it is appropriate for there to be textual similarity in the Methods as this is the same survey and hence using the same methods as in PONE22 with the important exception that this manuscript reports on a different dataset from the survey namely that on the prevalence and associative factors relating to the responsible research practices.*

**Methods: Could the authors check the part starting with " The explanatory factors scales were based..."? The second sentence seems to talk specifically about QRPs, and might not be applicable for this paper.**

*Author Response: This is making reference to the two explanatory factor scales namely, likelihood of QRP detection by collaborators and likelihood of QRP detection by reviewers. As such they are not making reference to the PONE22 QRP paper but to two of the twelve explanatory factor scales which are named as such.*

**This is very minor, but the text switched between "behavior" and "behaviour" and its derivatives.**

*Author Response: Thank you for pointing this out. We have checked the manuscript to ensure behavior is spelled consistently.*
